# Serial Living Solid Organ Donation: An Ethical Analysis

**DOI:** 10.1111/jep.70192

**Published:** 2025-06-29

**Authors:** Richard C. Armitage

**Affiliations:** ^1^ Academic Unit of Population and Lifespan Sciences School of Medicine, University of Nottingham, Clinical Sciences Building, Nottingham City Hospital Campus Nottingham

## Abstract

**Introduction:**

It is possible in many countries to not only become a living solid organ donor, but to become a serial living solid organ donor, a process in which an individual subsequently donates a liver lobe after donating a kidney, or vice versa. The major ethical issues that surround uncompensated living single solid organ donation (the doctor's duties to respect autonomy, of beneficence, and of non‐maleficence) have been well described, and this process is generally considered ethically permissible if the donor has sufficient health, and if their decision is voluntary, fully informed, and made in the absence of coercion. However, the landscape of ethical issues pertaining to serial living solid organ donation has so far gone unexamined.

**Methods:**

This paper conducts an ethical analysis, using the ethical framework of Principlism, of the ethical issues that surround serial living solid organ donation.

**Findings:**

Serial living solid organ donation not only repeats the ethical issues that pertain to single organ donation, but also compounds some of them. Respect for autonomy in serial donation is challenged by uncertainty of the long‐term risks of serial donation, and serial donors potentially face an increased risk of coercion from those in need of an organ and other third parties. The removal of a second healthy organ in serial donation poses greater risk to non‐maleficence than single organ donation because the enduring effects of the previous surgery increase surgical risk. The effect of serial donation on beneficence is currently unknown. Serial donation also generates the potentially novel ethical issue of the donation being motivated by pathological altruism (the act thereby being inspired by selfish concerns), which threatens autonomy, non‐maleficence, and beneficence.

**Discussion:**

Research is required to understand the long‐term risks to physical health and psychological wellbeing of serial donation to promote autonomy, non‐maleficence, and beneficence. Additionally, the understanding of pathological altruism as a motivating factor for living organ donation should be increased, and the psychological assessment of potential living donors should be vigilant to detect this motivation.

## 1 Introduction

It is possible in many countries for living individuals to donate a solid organ in a process known as living solid organ donation. For example, in the UK, living individuals are able to donate a solid organ – either a kidney or a liver lobe – through the National Health Service (NHS) [1]. Between 01 April 2023 and 31 March 2024, 938 living individuals donated a solid organ in the UK: 907 (96.7%) of these donated a kidney, and 31 (3.3%) donated a liver lobe [2].^1^

**Figure 1:**
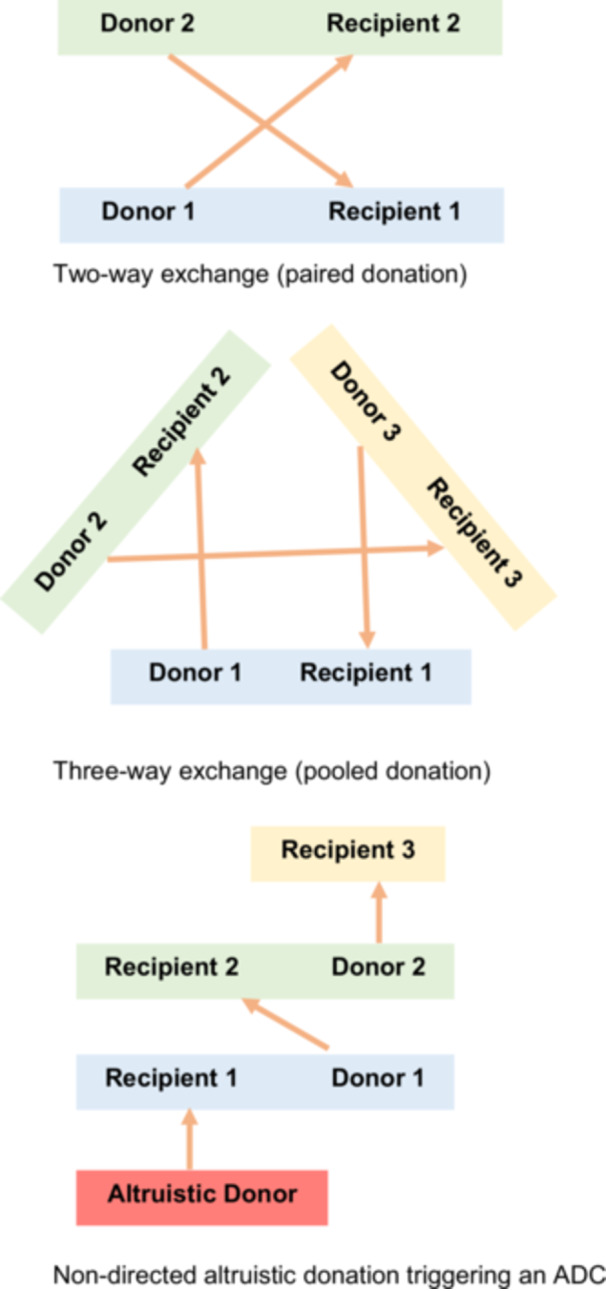
Two‐way exchange (paired donation). Three‐way exchange (pooled donation). Nondirected altruistic donation triggering an ADC.

It has been noted in the UK [5] and other countries in which living solid organ donation takes place [6, 7, 8, 9, 10, 11] that the availability of living solid organ donation might provide the opportunity to become ‘serial donors’ in which an individual subsequently donates a liver lobe after donating a kidney, or vice versa (this practice is alternatively referred to as ‘sequential donation’ or ‘repeat donation’). While publicly available data regarding serial donation is scarce, the number of individuals who become serial donors appears to be very low internationally: for example, between 1994 and 2023, there were 220 serial donors in the United States (the most common sequence was kidney then liver lobe donation [*N* = 158, 71.8%], followed by liver lobe then kidney donation [*N* = 48, 21.8%]) [12]; and, between 2007 and 2018 in Turkey, 5 living liver lobe donors subsequently donated a kidney [9].

The major ethical issues that surround uncompensated living solid organ donation have been well described [13, 14, 15], the most salient of which are respect for autonomy, beneficence, and non‐maleficence. However, the landscape of ethical issues pertaining to serial living solid organ donation has not been examined. This paper aims to conduct this ethical analysis by using the ethical framework of Principlism. The paper will focus on the serial donation of kidneys and liver lobes because these are the organ types that are serially donated most frequently (although the serial donation of other organ types, such as lung then kidney, kidney then pancreas, and liver then intestine, sometimes also takes place) [12].

## 2 Ethical Issues Surrounding Living Solid Organ Donation

Living solid organ donation is generally considered ethically permissible if the donor has sufficient physical and psychological health (such that donation is unlikely to cause substantial long‐term morbidity, or death, to the donor), and their decision is voluntary, fully informed, and made in the absence of coercion [5, 16, 17]. Accordingly, this prohibits the ethical donation of solid organs from children, and also from adults who lack capacity to make such impactful decisions.

Principlism is the normative ethical framework of professional ethics that was devised by Beauchamp and Childress in 1979 [18]. This framework, which was designed to aid ethical decision‐making in healthcare contexts, comprises of four basic and universal ethical principles that state prima facie moral obligations that are equally important for doctors in the provision of patient care. The Four Principles are the duties of respect for autonomy, beneficence, justice, and non‐maleficence. Since their introduction, the Four Principles have become the primary method for the teaching and evaluation of medical ethical dilemmas in healthcare contexts and [19], due to their strong support [20, 21, 22, 23], are still widely used today.

It is important to note that the Four Principles are only prima facie moral obligations, meaning they are not uncompromising duties that must always be upheld. Rather, these obligations are often in tension with one another, such that doctors must carefully and appropriately balance them for their actions to be ethically permissible.

### 2.1 Autonomy

Autonomy is the principle that individuals have the right to make decisions, hold views, and undertake actions based on their personal views and values. Broadly speaking, a person is autonomous if they govern their own decisions and actions. Accordingly, autonomy requires the doctor to respect the patient's capacity for self‐determination, and their ability to make independent decisions about their life in the absence of undue pressure, solicitation or coercion. For patients to make truly autonomous choices their consent to make those choices must be valid, which requires them to have access to and understand the relevant information pertaining to each choice. With regard to living solid organ donation, the duty to respect autonomy requires doctors to provide potential donors with the necessary information to make an informed decision regarding donation, and to ensure that the individual's wish to donate is not the result of their being subjected to undue pressure, solicitation or coercion. In the UK, a mandatory interview with an Independent Assessor (who acts on behalf of the Human Tissue Authority) is required of all potential solid organ donors as a means to identify and protect the potential donor against any undue pressure, solicitation or coercion [24, 25]. Independent Living Donor Advocates play a similar role in the US [26].

### 2.2 Non‐Maleficence

Non‐maleficence requires doctors, through their medical interventions, to avoid causing intentional and unnecessary harm to patients. The process of living solid organ donation by definition reduces the health of the donor as it removes from them a healthy and fully functioning organ that supports their health. Non‐maleficence, therefore, is in tension with the duty to respect the autonomous decision of potential living donors. It is generally viewed that, in potential living solid organ donors who are deemed to be psychologically and psychiatrically well, who have a sufficient degree of physical health [5, 19], and whose decision is voluntary, fully informed, and made in the absence of coercion, the duty to respect their autonomy should prevail and they should be allowed to become living donors.

### 2.3 Beneficence

Beneficence requires doctors to act for the benefit of the patient, such as preventing or removing harm, or the active promotion of some good, such as health. While living solid organ donation allows foreseeable harm to be caused to the donor (thereby violating beneficence), both living kidney donation [27, 28, 29] and living liver lobe donation [30, 31] are known to often improve the psychological wellbeing of the donor, meaning they might in fact serve to promote beneficence.

Accordingly, in living solid organ donation, the doctors’ duties to respect autonomy, of beneficence, and of non‐maleficence are in tension with one another. This process is generally considered ethically permissible if the donor has sufficient physical and psychological health (such that donation is unlikely to cause substantial long‐term morbidity, or death, to the donor), and their decision is voluntary, fully informed, and made in the absence of coercion.

## 3 Compounded Ethical Issues Surrounding Serial Living Solid Organ Donation

Once a solid organ has been donated by a living individual, and the individual forms an intention to donate a subsequent organ and, as such, become a serial solid organ donor, the ethical issues of respect for autonomy, non‐maleficence, and beneficence are once again raised. However, rather than being merely repeated in relation to the second donation, some of these ethical issues are in fact compounded in this context.

### 3.1 Autonomy

Like in the donation of the first solid organ, the duty to respect autonomy requires doctors to provide potential serial donors with the necessary information to make a decision regarding donation, and to ensure that the individual is not being subjected to undue pressure, solicitation or coercion. Meeting this duty poses at least two challenges that are greater than those raised by the first organ donation: first, morbidity and mortality data regarding serial living solid organ donation are sparse and incomplete (where they are reported, these data are primarily presented in single centre studies), while no long‐term studies have examined the long‐term risks of donating more than one solid organ sequentially [32, 33]. This is largely explained by the relatively small total number of serial organ donors, and lies in contrast to the morbidity and mortality data pertaining to single organ donors, which is well understood due to the much greater total number of such donors (e.g., the perioperative mortality is < 0.03% for living kidney donation [34], and 0.3% for living liver lobe donation) [35]. Accordingly, the paucity of long‐term outcome data for serial organ donors limits the extent to which the decision to become a serial organ donor can be informed, which might be considered to limit the autonomy with which such donors make their decision. However, an absence of complete information regarding the risk of serial donation does not make a decision to take that risk inherently nonautonomous. To promote the potential donor's autonomy, therefore, the doctor's duty is to ensure the donor is fully informed of the incomplete nature of the relevant information, and to ensure they understand the resulting uncertainty regarding the long‐term risks of becoming a serial donor. A decision made in the full understanding of this uncertainty is not inherently nonautonomous, and may in fact be entirely autonomous, since the donor is fully informed of all the available, albeit scarce, information.

Second, the risk of coercion might be greater in individuals who have already donated a solid organ than in those who have not, because their previous donation revealed them to be the kind of person who is both generally willing, and physically and psychologically able, to donate a solid organ. Such individuals’ previous donations might, therefore, increase the risk of their being targeted by undue pressure, solicitation or coercion to donate a second organ from two main sources: first, from those in need of a solid organ donation, such as a family member or friend who has end‐stage renal or liver failure (those in need of a liver lobe donation might pressure those who have previously donated a kidney to subsequently donate a liver lobe to them, while those in need of a kidney donation might pressure those who have previously donated a liver lobe to subsequently donate a kidney to them i.e. directed donation); second, from third parties who are not themselves in need of a solid organ donation but who recognise the donor as a virtuous individual due to their first organ donation, and who signal to the donor that becoming a serial donor is required of them for that virtue to continue (i.e. nondirected donation). While no evidence is available that attests to the prevalence of these potential sources of coercion in serial donation (qualitative studies with both serial donors and single organ donors who considered but did not proceed with serial donation are required to establish this), these scenarios are plausible and must be guarded against to maintain the donor's autonomy.

### 3.2 Non‐Maleficence

Like in the donation of the first solid organ, the duty of non‐maleficence requires doctors to avoid causing intentional and unnecessary harm to patients. The removal of a second healthy organ from a serial donor poses greater risk to the duty of non‐maleficence than is posed by the donation of the first organ. This is because the enduring effects of the previous surgery in which the first organ was removed for donation increase the surgical risk in the second donation. This is largely explained by the presence of adhesions, which are present in the second surgery as a result of the first and pose increased surgical risk [36]. Accordingly, the perioperative mortality in the second surgery in serial donation is likely to be greater than that of the same surgery that takes place in single donation (< 0.03% in kidney donation and 0.3% in liver lobe donation) [37, 38]. In addition, partial hepatectomy (which is necessary for liver lobe donation) is known to predict renal insufficiency [39, 40], the risks of which are likely to be greater in liver lobe donors who have previously donated a kidney because they possess only half their total prior‐donation renal tissue.

### 3.3 Beneficence

Like in the donation of the first solid organ, the duty of beneficence requires doctors to act for the benefit of the patient, such as preventing or removing harm, or the active promotion of some good, such as health. While living kidney donation and living liver lobe donation are known to often improve the psychological wellbeing of the donor, and therefore might serve to promote beneficence, the aforementioned paucity of long‐term outcome data in serial donation means the impacts of serial donation on the psychological wellbeing of such donors is unknown. While serial donation might repeat or even compound the positive psychological impacts of single organ donation, it is possible that it might harm the psychological wellbeing of the donor, potentially as a consequence of the greater risk of physical harm of serial donation versus single organ donation. Accordingly, the effect of serial donation on the duty of beneficence is unclear, and studies that examine the impact of serial donation on these donors’ psychological wellbeing is required to establish this.

## 4 A Novel Ethical Issue Surrounding Serial Living Solid Organ Donation

While serial living solid organ donation has been shown to not only repeat the ethical issues of single living solid organ donation, but to compound some of them, it also generates a novel ethical issue that is not necessarily raised when a single organ is donated.

The primary expressed reason for individuals choosing to become living solid organ donors is a desire to help the recipient (this is the case in both donations to known individuals and those to strangers) [36, 37]. However, no studies have been conducted that explore the reasons for why previous living solid organ donors subsequently choose to become serial donors. While it is entirely possible that the same desire to help the recipient that motivates single organ donation also inspires serial organ donation, there is the potential for a pathological form of altruistic behaviour to be motivating such donors.

Pathologically altruistic acts are fundamentally motivated by selfish concerns about a fear of rejection and a fear of losing emotional intimacy stemming from low self‐esteem [38, 41]. Pathological altruism is associated with aspects of vulnerable narcissism (including the need for admiration) and a fear of rejection, losing emotional contact, and losing control [38]. Individuals scoring high on a pathological altruism scale are substantially more likely to report that they help others to avoid rejection and criticism, gain approval, and please others [38].

Pathological altruism might motivate an individual to become a living solid organ donor [42, 43]. However, it is reasonable to expect the prevalence of this driver to be greater among serial donors than single donors because the previous donation of serial donors revealed them to be the kind of person who is both willing and able to donate a solid organ, meaning the serial donor might surmise that others expect of them a second organ donation, which they subsequently carry out to avoid rejection and gain approval. While no data are available regarding the prevalence of pathological altruism as a motivating factor for single or serial living solid organ donation, the fact that it is a known potential motivator for living donation means there is a clear possibility for it to also motivate serial donation. This is of ethical importance because the influence of pathological altruism might undermine the donor's autonomy if, for example, their pathological altruism was sufficiently powerful to override any inhibiting factors, such as the wish not to be exposed to the substantial risks inherent to donation. This is in addition to pathological altruism‐driven donation subjecting the donor to the consequential harms of donation, thereby threatening non‐maleficence (the impact on beneficence on such donors is unknown due to the lack of long‐term outcome data in serial donation). It is, therefore, important that the psychological assessment of potential donors (both single organ donors and, especially, serial organ donors) that is integral to the process of ethically permissible living organ donation [5, 30] is conducted with high vigilance to detect the possible presence of pathological altruism as a motivating factor for donation.

## 5 Conclusion

While the number of individuals who become serial living solid organ donors is very low internationally, it is possible to become such a donor in many countries. The major ethical issues that surround uncompensated living solid organ donation have been well described, and are the doctor's duties to respect autonomy, of non‐maleficence, and of beneficence. While these duties are in tension with one another in living solid organ donation, this process is generally considered ethically permissible if the donor has sufficient health (such that donation is unlikely to cause substantial long‐term morbidity, or death, to the donor), and their decision is voluntary, fully informed, and made in the absence of coercion.

When a living solid organ donor forms an intention to become a serial living solid organ donor, the ethical issues of respect for autonomy, non‐maleficence, and beneficence are raised once again, but some are also compounded. Respect for autonomy in serial donation faces at least two challenges that are greater than those raised by the first donation: first, the uncertainty regarding the long‐term risks of becoming a serial living solid organ donor (due to the paucity of long‐term outcome data) might reduce the ability of a potential serial donor to make a fully informed choice; second, the risk of coercion, both from those in need of a solid organ donation and from third parties who are not themselves in need of an organ, might be greater in individuals who have already donated a solid organ than in those who have not. The removal of a second healthy organ from a serial donor poses greater risk to the duty of non‐maleficence than is posed by the donation of the first organ because the enduring effects of the previous surgery increase the surgical risk in the second donation. Finally, the effect of serial donation on the duty of beneficence is unknown as there is an absence of long‐term data on the risks of serial donation. In addition to these compounded ethical issues, serial organ donation also generates the novel ethical issue of the donation being motivated by pathological altruism – which threatens the donor's autonomy and non‐maleficence – that is not necessarily raised when a single organ is donated.

## 6 Recommendations for Policy and Practice

For the ethical issues surrounding serial living solid organ donation to be mitigated, both quantitative and qualitative studies are required to understand the long‐term risks of serial donation on physical health and psychological wellbeing, such that a decision to become a serial donor can be more thoroughly informed (thereby promoting respect for autonomy), and the impact on the duties of non‐maleficence and beneficence can be more greatly understood. In addition, research is required to identify the prevalence of pathological altruism as a motivating factor for living solid organ donation, particularly among serial donors, such that the psychological assessment of living donors can be appropriately vigilant to detect this reason for donation.

## Conflicts of Interest

RA is a non‐directed altruistic kidney donor.

## Transparency Declarations

RA is a non‐directed altruisitic kidney donor.

## Data Availability

The author has nothing to report.

## Endnotes

1 Solid organ donation is termed ‘altruistic’ if two conditions are satisfied: first, the donor has no prior relationship with the recipient; second, the donor has not agreed to donate an organ for a loved one to receive an organ from another donor in return [3]. Altruistic donation is either directed or nondirected: in directed altruistic donation, the organ is donated to a specific individual with whom the donor has no prior relationship (such as the subject of a media campaign); in nondirected altruistic donation, the organ is donated to an unspecified individual with whom the donor has no prior relationship [4]. Living kidney donations can be ‘shared’ across the UK through the UK Living Kidney Sharing Scheme (UKLKSS) [3]. This contains linked donor–recipient pairs which consist of an individual in need of a kidney (recipient) and another individual (a loved one) who is willing to donate one (donor), but the pair's tissue incompatibility prohibits a donation between them. Through the UKLKSS, incompatible linked donor‐recipient pairs are ‘matched’ with other such pairs that, in some combination, are collectively compatible for kidney sharing. In Paired‐Pooled Donations, two‐way (paired donation) sharing occurs between two linked pairs, while three‐way (pooled donation) sharing occurs between three linked pairs. Although such donations are undoubtedly virtuous, and despite the donor having no prior relationship with the recipient, they are not described as ‘altruistic’ because the donor's loved one, with whom they have formed a linked donor–recipient pair, receives an organ in return. In Altruistic Donor Chains, individuals not in linked pairs who wish to donate a kidney without a loved one receiving one in return donate to a recipient in a linked donor‐recipient pair to trigger a chain of multiple donations. Such donations are considered to be ‘altruistic’ because the donor has no prior relationship with the recipient, and has not formed a linked donor‐recipient pair with a loved one. If tissue incompatibility prevents the formation of an Altruistic Donor Chain, individuals not in linked pairs who wish to donate a kidney without a loved one receiving one in return can donate to an unspecified individual without the formation of an Altruistic Donor Chain (see Figure 1). While there is currently no equivalent sharing scheme for living liver lobe donations in the UK (meaning no Paired‐Pooled Donations or Altruistic Donor Chains are possible for living liver lobe donations), donors are able to donate to unspecified individuals as nondirected altruistic donors. In these donations, donors have no prior relationship with the recipient, and the donor has not formed a linked donor‐recipient pair with a loved one.
